# 

**Published:** 1842-12-10

**Authors:** 


### CONTENTS OF No. 50.

Dr. C. H. Harris' Case of Congeuital Enlargement of the Parotid Gland, - - - - - - - - - 785

BIBLIOGRAPHICAL NOTICES: On Recent Improvements in Surgery. An Introductory Lecture to the Course on the Principles and Practice of Surgery, in Jefferson Medical College of Philadelphia. Delivered November 3, 1842. By Thomas D. Mütter, M. D., - - - - - - - - - 786

A Lecture Introductory to the Course of Obstetrics in Jefferson Medical College of Philadelphia. Delivered November 5, 1842. By C. D. Meigs, M. D., - - - - - - 788

ANALECTA: Dr. Stricker on the Hospitals of Italy, - - - - 792

Dr. Symonds on Therapeutics, - - - - - - 795

